# No evidence for a shift in pyruvate kinase PKM1 to PKM2 expression during tumorigenesis

**DOI:** 10.18632/oncotarget.278

**Published:** 2011-05-22

**Authors:** Katharina Bluemlein, Nana-Maria Grüning, René G. Feichtinger, Hans Lehrach, Barbara Kofler, Markus Ralser

**Affiliations:** ^1^ Max Planck Institute for Molecular Genetics, Berlin, Germany; ^2^ Research Program for Receptorbiochemistry and Tumormetabolism, Department of Pediatrics, Paracelsus Medical University, Salzburg, Austria; ^3^ Cambridge Systems Biology Centre and Department of Biochemistry, University of Cambridge, Cambridge, United Kingdom

**Keywords:** pyruvate kinase, proteomics, cancer metabolism, alternative splicing, Warburg effect

## Abstract

The Warburg effect describes the circumstance that tumor cells preferentially use glycolysis rather than oxidative phosphorylation for energy production. It has been reported that this metabolic reconfiguration originates from a switch in the expression of alternative splice forms (PKM1 and PKM2) of the glycolytic enzyme pyruvate kinase (PK), which is also important for malignant transformation. However, analytical evidence for this assumption was still lacking. Using mass spectrometry, we performed an absolute quantification of PKM1 and PKM2 splice isoforms in 25 human malignant cancers, 6 benign oncocytomas, tissue matched controls, and several cell lines. PKM2 was the prominent isoform in all analyzed cancer samples and cell lines. However, this PKM2 dominance was not a result of a change in isoform expression, since PKM2 was also the predominant PKM isoform in matched control tissues. In unaffected kidney, lung, liver, and thyroid, PKM2 accounted for a minimum of 93% of total PKM, for 80% - 96% of PKM in colon, and 55% - 61% of PKM in bladder. Similar results were obtained for a panel of tumor and non-transformed cell lines, where PKM2 was the predominant form. Thus, our results reveal that an exchange in PKM1 to PKM2 isoform expression during cancer formation is not occurring, nor do these results support conclusions that PKM2 is specific for proliferating, and PKM1 for non-proliferating tissue.

## INTRODUCTION

Malignant cell growth entails numerous metabolic changes. The so called ‘Warburg’ effect describes the decrease in respiration during tumor development, whereas glucose uptake and aerobic glycolysis, as well as lactate production increases [1-3]. The reason why cells undergo the Warburg effect are not entirely understood, but it is broadly assumed that the switching-off of the respiratory metabolism increases metabolic intermediates that are required for the synthesis of biological macromolecules [1, 2, 4]. This assumption is supported by the fact that stroma type cells that deliver metabolites utilized by the tumor for energy production also undergo this metabolic transition [5, 6]. Furthermore, as glycolytic fermentation circumvents oxidative phosphorylation in the respiratory chain, it avoids the release of superoxide from complex I and III, which could prevent oxidative stress [7]

Although important signaling cascades of cellular metabolism such as STAT3 and HIF-1α have been implicated in the regulation of the Warburg effect [8, 9], the mechanisms how it is initiated remain elusive. It has been reported that an exchange in the expression of PKM1 to PKM2, two alternative splice isoforms of the glycolytic enzyme pyruvate kinase (PK) [10, 11], is causative for the Warburg effect during tumorigenesis [10]. These two isoforms differ in a single exon, which facilitates binding of the glycolytic intermediate fructose 1,6 bisphosphate in PKM2 type PK. PKM1 is constitutively active, whereas PKM2 can switch between an active tetrameric and an incactive dimeric form [12].

It has been concluded from Western blot analysis of cancer cell lines (A549, H1299, 293T, HeLa, MCF10a) and Western blot/immunostaining of mammary gland tissue from MMTV-NeuNT mice that cancer development switches expression from PKM1 to PKM2 [10]. These conclusions were drawn from the comparison of PKM1/PKM2 expression in cancer cell lines with human muscle [10]. However, as protein expression is highly tissue dependent [9,10], and as earlier biochemical studies had reported that pyruvate kinase PKM2 is present in several healthy tissues [13], we re-investigated PKM1/PKM2 expression in tumors, taking into account tissue-matched controls.

Using an absolute quantification (AQUA) strategy with isotope labeled standards, we performed a comprehensive absolute quantification of PKM1 and PKM2 in several cancer tissue of different origin, benign tumors and cell lines, and their tissue matched controls. We found no evidence for an exchange of PKM1 to PKM2 expression during cancer formation. Cancers maintained the PKM isoform expression according to their tissue of origin.

## RESULTS

### Development of an absolute quantification (AQUA) method to quantify of PKM1 and PKM2 in cell extracts

**Figure 1:**
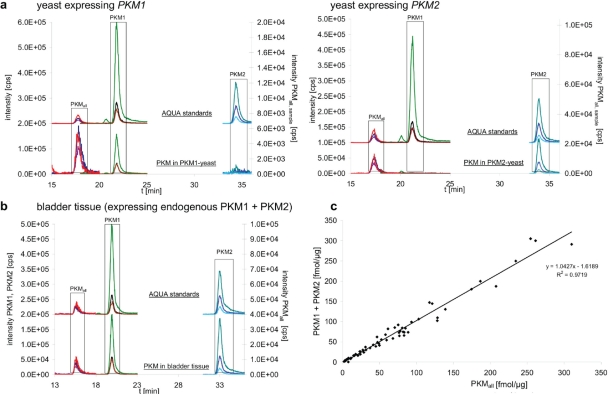
Absolute quantification of PKM1 and PKM2 splice forms in tissue extracts **a**. Yeast expressing human PKM1 (PKM1-yeast, left panel) and human PKM2 (PKM2-yeast, right panel) were analyzed by nanoflow liquid chromatography/multiple reaction monitoring (LC-MRM) to quantify a PKM1 and a PKM2 specific peptide as well as a peptide which is specific for both isoform (PKM_all_) (lower chromatograms). Matching heavy isotope labeled peptides (AQUA peptides) were included in every sample and used for quantification (upper chromatograms, please note that they are displaced on the Y axis for better illustration). The determined concentrations were 3.3 fmol/μg protein for PKM1, and 19.3 fmol/μg protein for PKM2 in yeast. **b**. Exemplary chromatogram for a human tissue sample, quantification of PKM1 and PKM2 in bladder tissue by LC-MRM. The analysis was performed as in (a). Absolute and relative values determined in human tissue are given in Table 1. **c**. Plot of the concentrations obtained for PKM1 plus PKM2 against the concentration of a peptide specific for both isoforms [PKM_all_]. The obtained concentrations show linear correlation (R² > 0.97)

We decided on an absolute quantification of PKM1 and PKM2 by mass spectrometry, since this technology circumvents the drawbacks that may result from the use of antibodies in semiquantitative westernblotting [14, 15] used in earlier studies [10]. As antibodies differ in affinity, similar band intensities obtained with different antibodies do not indicate similar concentration of their target proteins. In contrast, the AQUA strategy allows absolute quantification of a non-purified protein at physiological concentration [16] by spiking the samples with chemically synthesized, heavy-isotope labeled peptide standards (AQUA peptides) that match the proteolytic peptide of interest in sequence, but are distinguishable from the analyte by mass [14, 15, 17]. To assure accurate PKM quantification, and to detect a potential switch in PKM isoform expression, this analysis was conducted with three PKM specific isotope labeled peptides and on a hybrid ion trap/triple quadrupole mass spectrometer operating in MRM mode. We selected one peptide to be specific for PKM1 (PKM1_LFEELVR_), one peptide for PKM2 (PKM2_LAPITSDPTEATAVGAVEASFK_), and a third peptide specific for both forms (PKM_all ITLDNAYMEK_). We tested PKM1 and PKM2 quantification with these peptides on transgenic yeast expressing exclusively either human PKM1 (PKM1-yeast) or human PKM2 (PKM2-yeast). The yeast strains were generated by cloning human PKM1 and PKM2 cDNA into an expression vector and transformation into the yeast strain BY4741. Protein extracts were generated, separated by SDS-PAGE, in-gel digested with trypsin [18], supplemented with the AQUA peptides [15] and analyzed as described previously [19]. The three isotope labeled standards were detected in all samples (Fig 1A, upper chromatograms). In PKM1-yeast, the PKM1 specific and the PKM_all_ peptide were detected but not the PKM2 peptide. In contrast, the analysis of PKM2-yeast detected the PKM2 specific peptide and the PKM_all_ peptide, but not the PKM1 peptide (Fig. 1A, lower chromatograms). Thus, the PKM1 and PKM2 specific peptides were detected and allowed specific discrimination between the PKM isoforms.

**Table 1:** Absolute amount of PKM1 and PKM2 as well as relative PKM content in human tumors, control tissues and cell lines Concentrations are given in fmol per μg protein

Sample	PKM1 [fmol μg^−1^]	PKM2 [fmol μg^−1^]	PKM1 [%]	PKM2 [%]
Malignant tumors				
Renal cell carcinoma 1 (RCC1)^§^	3.2	62.8	4.8	95.2
Renal cell carcinoma 2 (RCC2)^§^	1.2	39.3	3.0	97.0
Renal cell carcinoma 3 (RCC3)^§^	3.1	139.4	2.2	97.8
Renal cell carcinoma 4 (RCC4)^§^	2.2	125.5	1.7	98.3
Bladder carcinoma 1 (BC1)	5.5	181.9	2.9	97.1
Bladder carcinoma 2	4.4	116.0	3.7	96.3
Bladder carcinoma 3	4.6	286.7	1.6	98.4
Bladder carcinoma 4	3.5	55.1	6.0	94.0
Hepatocellular carcinoma 1 (HCC1)	0.6	5.8	9.4	90.6
Hepatocellular carcinoma 2 (HCC2)	n.d.	10.3		100
Hepatocellular carcinoma 3 (HCC3)	0.5	45.2	1.1	98.9
Colorectal carcinoma 1 (CRC1)	1.4	129.1	1.1	98.9
Colorectal carcinoma 2 (CRC2)	1.0	110.2	0.9	99.1
Colorectal carcinoma 3	14.7	323.4	4.3	95.7
Lung carcinoma 1 (LC1)	3.1	80.5	3.7	96.3
Lung carcinoma 2 (LC2)	3.5	95.8	3.5	96.5
Lung carcinoma 3	0.8	47.0	1.7	98.3
Lung carcinoma 4	0.9	59.9	1.5	98.5
Lung carcinoma 5	1.3	49.8	2.5	97.5
Follicular thyroid adenoma 1	0.5	14.6	3.3	96.7
Follicular thyroid adenoma 2	0.7	32.5	2.1	97.9
Follicular thyroid adenoma 3	0.6	29.4	2.0	98.0
Follicular thyroid adenoma 4	2.3	43.9	5.0	95.0
Follicular thyroid adenoma 5	0.9	37.0	2.4	97.6
Papillary thyroid carcinoma 1	2.2	69.8	3.1	96.9
Benign tumors				
Renal oncocytoma 1^#^	1.4	128.1	1.1	98.9
Renal oncocytoma 2^#^	1.2	83.6	1.4	98.6
Renal oncocytoma 3	0.9	57.9	1.5	98.5
Thyroid oncocytoma 1 (TO1)	3.8	60.1	5.9	94.1
Thyroid oncocytoma 2 (TO2)	0.3	14.8	2.0	98.0
Thyroid oncocytoma 3	1.1	31.9	3.3	96.7
Control tissues				
Kidney 1 (RCC1)^§^	0.8	27.6	2.8	97.2
Kidney 2 (RCC2)^§^	0.8	24.8	3.1	96.9
Kidney 3 (RCC3)^§^	0.8	32.6	2.4	97.6
Kidney 4 (RCC4)^§^	0.7	33.8	2.0	98.0
Bladder 1 (BC1)	17.2	20.8	45.3	54.7
Bladder 2	30.3	46.7	39.4	60.6
Liver 1 (HCC1)	n.d.	5.2		100
Liver 2 (HCC2)	n.d.	15.2		100
Liver 3 (HCC3)	n.d.	14.4		100
Colon 1 (CRC1)	4.9	65.9	6.9	93.1
Colon 2 (CRC2)	2.8	70.1	3.8	96.2
Colon 3	9.7	38.3	20.2	79.8
Lung 1 (LC1)	1.3	23.9	5.2	94.8
Lung 2 (LC2)	0.8	15.2	5.0	95.0
Thyroid 1 (TO1)	0.8	11.7	6.4	93.6
Thyroid 2 (TO2)	0.8	11.9	6.3	93.7
Thyroid 3	0.5	9.7	4.9	95.1
Thyroid 4	1.4	22.3	5.9	94.1
cancer cell lines				
60138 A1 [Tumor associated fibroblasts, breast] (60161 B1)	21.0	88.7	19.1	80.9
87442 A1 [breast cancer associated fibroblasts]	17.4	47.6	26.8	73.2
A459-1 [lung carcinoma]	3.1	302.0	1.0	99.0
A459-3 [lung carcinoma]	3.2	297.1	1.1	98.9
HCT [Human colon tumor]	3.1	16.4	15.9	84.1
HEK-1 [transf., embryonic kidney]	2.3	79.9	2.8	97.2
HEK-2 [transf., embryonic kidney]	2.1	83.2	2.5	97.5
HEK-3 [transf., embryonic kidney]	2.6	59.6	4.2	95.8
HeLa-1 [cervix adenocarcinoma]	2.4	144.9	1.6	98.4
HeLa-2 [cervix adenocarcinoma]	2.4	142.1	1.7	98.3
HEP-1 [hepatocellular carcinoma]	8.1	241.8	3.2	96.8
HEP-2 [hepatocellular carcinoma]	6.5	193.4	3.3	96.7
MCF 7 [breast epithelial adenocarcinoma]	1.3	66.7	1.9	98.1
MDA MB-415 [Breast epithelial adenocarcinoma]	0.6	83.8	0.7	99.3
SPH 77-1 [small cell lung cancer]	0.5	3.8	11.6	88.4
SPH 77-2 [small cell lung cancer]	n.d.	0.5		100
other & control cell lines				
60161 B1 [breastcancer adjacent fibroblast] (60138 A1)	32.7	147.5	18.1	81.9
37098 B1 [breastcancer adjacent fibroblast]	10.6	30.8	25.6	74.4
MCF 10A [Breast epithelial cell line]	1.0	72.9	1.4	98.6
MCF 12A [Breast epithelial cell line]	2.1	89.9	2.3	97.7
The abbreviations given in brackets for the control tissues refer to the matched tumor tissues. §The renal cell carcinomas and control tissues have been analyzed in a previous study [25], Renal oncocytoma 1 was case 6, Renal oncocytoma 2 was case 14 in [26].				

### Quantification of PKM1 and PKM2 in human tissue and cancer

**Figure 2:**
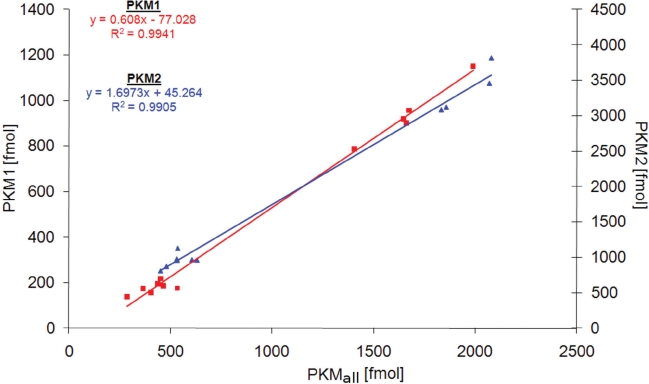
Accuracy of PKM1 and PKM2 quantification Differently concentrated digests of PKM1-yeast (n = 11) and PKM2-yeast (n = 12) were injected, and the peptides PKM1, PKM2, and PKM_all_ quantified as well as their corresponding AQUA standards analyzed by LC-MRM. Shown is a correlation plot of the concentration of the specific peptide (PKM1 or PKM2) and the PKM_all_ peptide, concentrations are given in absolute values (fmol).

To study PKM1 and PKM2 expression before and after cancer development, we analyzed 25 human malignant cancers, 18 tissue-matched controls, 12 cancer cell lines, 4 non-cancer cell lines and 6 benign oncocytomas. In 15 cases (12 malignant cancers, 2 benign tumors, 1 cell line), matched unaffected tissue from the affected individual was available. As described above, the samples were supplemented with the heavy-isotope labeled standards, and the three peptides quantified by multiple reaction monitoring on the QTRAP mass spectrometer. Peptides corresponding to both PKM1 and PKM2 were detected in tissues, cell lines and controls (Fig 1b). To test if the chosen peptides gave consistent results in quantifying PKM1 and PKM2, we plotted the obtained concentration values of PKM1 plus PKM2 versus the quantity obtained for the PKM_all_ peptide which is characteristic for both PKM alternative splice isoforms. The quantities showed with R² = 0.97 a linear correlation, confirming that the chosen peptides were suitable for PKM quantification (Fig 1c). In addition, we tested the reproducibility of PKM1 and PKM2 quantification by performing multiple injections for PKM1 and PKM2 yeast samples at different concentration. Linear regression was demonstrated by a R² of 0.994 for PKM1 and 0.990 for PKM2 (Fig 2), and thereby representing reliability of the quantification experiments.

### PKM2 dominates in cancer and tissue-matched controls

We found that PKM2 was the predominant PKM isoform in all human cancer cell lines (Table 1), which is in agreement with the earlier results obtained by Western blotting [10]. MCF10a, HeLa, A459 and a HEK-cell line (HEK293) were included in both studies, the AQUA analysis revealed that PKM2 accounted for 98.6% (MCF10a), 98.4% (Hela), 98.9-99.0% (A459) and 95.8%-97.5% (HEK293) of total PKM. Thus mass spectrometry gave similar results as Western blotting, but the LC-MRM technology was more sensitive as PKM1 was clearly detectable in all samples, even at the lower femtomol range.

PKM1 and PKM2 quantification in further cell lines and malignant cancer samples confirmed the conclusion of PKM2 being the prominent PKM in all analyzed malignant cancer types: PKM2 accounted for a minimum of 94% of total PKM in all 22 malignant cancers, and other cell lines (Table 1). However, we found that PKM2 was also the dominant isoform in matched control tissue and slowly proliferating tumors. PKM1 and PKM2 were quantified in 18 healthy human tissues, and four non-cancer derived cell lines. In healthy kidney, lung, liver, and thyroid tissue, PKM2 accounted for a minimum of 93% of total PKM, for 79.8%-96.2% of PKM in colon, and 54.7%-60.6% of PKM in bladder. A similar picture was seen also in the oncocytoma samples. In these slow growing tumors, PKM2 accounted for 94.1-98.9% of total PKM. In table 1, the matching control/cancer tissue of the same individual is indicated in brackets. A switch in the expression from PKM1 to PKM2 during cancer development was not observed in any case. Only in a single case (bladder carcinoma) the control tissue had a much lower relative amount of PKM2 (54.7%) then the cancer sample (97.1% PKM2), but the change in the percentage resulted predominantly from an up regulation of the PKM2 isoform from 20.8 fmol in the bladder control to 181.9 fmol in the cancer, and not from a switch in alternative splicing.

In general, total PKM was expressed at a higher level in the cancer as in the control tissue. For instance, the average renal cell carcinoma tissue had 94.2 fmol PKM/μg protein, control kidney 30.5 fmol. This corresponds to a three-fold upgregulation in the absolute values. However, PKM1 and PKM2 were equally affected (an increase of 3.1 fold for PKM1 and 3.1 fold for PKM2).

## DISCUSSION

This study addresses a common misinterpretation of the finding that pyruvate kinase PKM2 is expressed in cancer cells. Pyruvate kinase is the terminal enzyme in glycolysis. It converts phophoenol-pyruvate to pyruvate, a reaction which yields one molecule of ATP, therefore it accounts for glycolytic energy production. The PK product pyruvate is then converted to lactate which is excreted, or enters the mitochondrial citrate cycle. Humans possess four isoforms of pyruvate kinase, an L and R form, present in liver and red blood cells, and the M1 and M2 form, which were originally identified in muscle [11, 13]. Furthermore, based on the data generated with cell lines, a switch of PKM1 to PKM2 during development of cancer was postulated [10]. The results presented here, which base on a quantitative analytical platform that allowed the investigation of multiple samples and tissue-matched controls, challenges these conclusions. Quantitative analysis of PKM1 and PKM2 expression in different cancers and matched control tissue showed that a switch in the expression between these alternative splice isoforms is not associated with tumor development. According to these results PKM2 is not specific for rapidly proliferating tissue, nor tumors. However, the results agree that total PKM is up-regulated in cancer, which matches the observation of a high glycolytic activity of cancer cells.

Our findings prompt for a re-examination of the conclusion drawn in earlier studies [10, 20], and subsequent investigations that are based on these reports. The absolute values, presented here, revealed that the total (PKM1+PKM2) concentration varies highly between tissues, for example lung tissue contained 12.5 - 16.0 fmol PKM/μg protein, whereas in unaffected colon tissue 70.8 -72.9 fmol PKM/μg protein were found. This underlines the requirement of tissue matched controls for analyzing a change in the expression of PKM isoforms. Our results show that the nature of the tissue is the prime determinant of the expressed PKM isoform. For instance, fibroblasts maintained a higher relative PKM1 as other cell lines, irrespective if they were transformed or not (Table 1). This fact might also explain the higher PKM1 content in healthy bladder tissue, as muscle dominates unaffected bladder tissue, and PKM1 was the prominent PKM isoform in muscle [10].

In light of these results it has to be considered that the high concentrated PKM2, although possessing a lower catalytic activity as PKM1 [21], is responsible for most PKM activity in most healthy and cancer tissue. Thus, its exchange by a PKM1 isoform at its endogeneous level would cause a reduction in total PK activity, whereas the observed up-regulation an increase in PK activity. Following this way of thinking, tumors of PKM expressing tissues can possess higher pyruvate kinase activities as their matched controls.

Although the new results require that the current model of glycolysis regulation in cancer has to be re-examined, they do not exclude the possibility that a change in PK activity due to posttranslational modifications of PKM2 is involved in regulating respiratory metabolism. PKM2 can change from its dimeric into a tetrameric form [12], and electrophoretic shift variants point to different post-translationally modified versions of PKM2 [22]. The results are consistent with other investigations which demonstrate that phosphorylation can tune PKM2 activity in cancer [23]. Thus dynamic tuning of PKM2 activity, but not an exchange of PKM1 to PKM2 isoform expression, might be responsible for the tumor cell's Warburg effect.

## METHODS

### Plasmid generation

Plasmids encoding pyruvate kinase PKM1 and PKM2 were generated by amplifying PKM1 from human fetal brain cDNA and PKM2 from cDNA of a pool of twenty adult tissues (Invitrogen) by PCR with primers 5'-GAGAATTCATGTCGAAGCCCCATAGTG -3' and 5'-GAGTCGACTCACGGCACAGGAACAAC -3'. PCR products were ligated into centromeric yeast plasmids containing the *TEF1* promoter (p413TEF) [24]. The plasmids were verified by restriction digest and re-sequencing.

### Sample preparation and analytical method

Human tissue were processed as described earlier [25]. In brief, frozen tissues were cut into 5 μm thick sections with a cryomicrotome at – 20°C. 50 – 100 mg tissue were transferred in 10 – 20-fold volume of SEKT buffer (250 mM saccharose; 2 mM EGTA, 40 mM KCl; 20 mM TRIS; pH 7.4). The samples were homogenized with Potter-S-Homogenisator on ice and centrifuged 10 min at 600g. The supernatant was aliquoted and stored at -70°C

Protein samples from yeast carrying p413TEF *-PKM1* or p413TEF-*PKM2* and human cancer and control tissues were separated on a 10% SDS-PAGE gel and the region corresponding to the mass range 50-70 kDa was excised. Those gel pieces were then subjected to an in-gel tryptic digest, adapted from Kaiser et al. [18]. The AQUA peptide mixture (20 μl) containing all three labeled peptides was spiked to the samples after the digest. The LC-MRM analysis was performed on a nanoLC (Eksigent, Ultra 2D) coupled online to a hybrid triple quadrupole/ion trap mass spectrometer (AB/SCIEX, QTRAP5500) as described earlier [19]. In brief, as mobile phase 0.1% formic acid in water (A) and 0.1% formic acid in acetonitrile (B) were used. After trapping the analytes and standards on a trap column (ReproSil pur, C18-AQ, 5 μm, 0.15 x 10 mm), they were eluted onto a RP-analytical column (Agilent, Zorbax SB300-C18; 3.5 μm, 0.75 x 150 mm). Separation was achieved by applying a linear gradient starting at 15% B and going up to 30% B within 30 min. The acetonitrile content was then increased to 95% within the next 10 min and kept at that level for 15 min before returning to the starting conditions. The tryptic peptide for PKM1 (LFEELVR) and its isotope labeled analogue (LFEE[LC13N15]VR) were monitored on the MRM transitions resulting from 2y4; 2y5 and 2y6 fragmentation. The tryptic peptide LAPITSDPTEATAVGAVEASFK, specific for PKM2, and its isotope labeled analogues (LAPITSDPTEATAVGAVEAS[FC13N15]K) were monitored on the MRM transitions attributed to 3y8; 3y9 and 3y10 fragment ions. The tryptic peptide ITLDNAYMEK, obtained from both PKM isoforms, and the corresponding isotope labeled peptide (IT[LC13N15]DNAYMEK) were detected on MRM transitions deriving from 2y6, 2y7 and 2y8 fragmentation. Using the peak area of the PKM_all_ peptide in the yeast sample as reference, we calculated a correction factor of 2.1 for the peak areas measured for the PKM1 specific peptide, and 0.6 for PKM2 peptide. Every sample injection was followed by an acetonitrile injection to exclude sample carry over. The identity of the quantified peptides was confirmed by collecting of MS/MS spectra on the QTRAP operating in iontrap mode.

## ETHICS

Tissue samples were kindly provided by the Biobank of the Medical University Graz, the Institute of Pathology and the Department of Urology, Paracelsus Medical University Salzburg. All tissues were frozen and stored in liquid nitrogen within 20 minutes after surgery. Tumor cell content and cellular composition of samples were evaluated using hematoxylin-eosin-stained frozen sections. All analyzed cancer samples had a tumor cell content of over 80%. Matching normal tissue was available for samples given in Table 1. The study was performed according to the Austrian Gene Technology Act. Experiments were performed in accordance with the Helsinki declaration of 1975 (revised 1983) and the guidelines of the Salzburg State Ethics Research Committee being no clinical drug trial or epidemiological investigation. All patients have signed an informed consent concerning the surgical removal and therapy of the tumors. Furthermore, the study did not extend to examination of individual case records. The anonymity of the patients has been ensured.
